# Vaccination Attitude and Communication in Early Settings: An Exploratory Study

**DOI:** 10.3390/vaccines8040701

**Published:** 2020-11-20

**Authors:** Noemi Mereu, Alessandra Mereu, Alessandra Murgia, Arianna Liori, Michela Piga, Federico Argiolas, Graziella Salis, Simonetta Santus, Carmela Porcu, Paolo Contu, Claudia Sardu

**Affiliations:** 1 Department of Medical Science and Public Health, University of Cagliari, University Campus of Monserrato, 09125 Cagliari, Italy; n.mereusalute@gmail.com (N.M.); amereu@unica.it (A.M.); murgiaale@gmail.com (A.M.); arianna.liori@unica.it (A.L.); michelina.miky@hotmail.it (M.P.); pcontu@unica.it (P.C.); 2 Sardegna, Regional Health Authority, 09123 Cagliari, Italy; federico.argiolas69@aob.it; 3 Sardegna, Agency for Health Care, ASSL Nuoro, Via Piemonte, Macomer, 08015 Nuoro, Italy; grazsalis@tiscali.it; 4 Sardegna, Agency for Health Care, ASSL Cagliari, Via Sonnino, 09125 Cagliari, Italy; simonetta.santus@atssardegna.it; 5 University Hospital of Cagliari, 09125 Cagliari, Italy; carmela.porcu@libero.it

**Keywords:** vaccination attitude, vaccine hesitancy, new-born babies’ mothers, early communication, prenatal courses, birth centre

## Abstract

*Background:* This study assesses attitudes towards vaccination in mothers of new-born babies and explores its association with different exposures to communication. *Methods:* Data were collected through questionnaires administered by means of interviews. *Results:* Data highlighted that 20% of mothers showed an orientation towards vaccine hesitancy. As for the reasons behind the attitude to vaccine hesitancy, data showed that concern is a common feature. As for the different exposures to communication, 49% of mothers did not remember having received or looked for any information about vaccination during pregnancy and post-partum; 25% stated they received information from several healthcare and non-healthcare sources; 26% declared having received or looked for information by means of healthcare and non-healthcare sources, as well as having taken part in a specific meeting during antenatal classes or at birth centres. The attitude towards vaccine hesitancy tends to reduce as exposure to different communication increases. *Conclusions:* This study supports the hypothesis that participation in interactive meetings in small groups focused on vaccination during the prenatal course or at the birth point may act as an enabling factor contributing to a decrease in the tendency to experience vaccine hesitation.

## 1. Introduction

Although vaccination programmes are one of the major preventive measures to control communicable diseases, decline of trust in vaccines is spreading and coverage of childhood immunization has decreased in several high-income countries [1,2,3,4]. 

In Europe, in order to contrast the spread of vaccines’ rejection and/or delay, countries such as Italy and France enacted laws that make several vaccinations at the paediatric age mandatory, and other countries are also considering introducing immunization mandates. Childhood mandatory vaccination is a useful strategy when a country faces declining vaccination rates; however, it should be accompanied by strategies to spread the “culture of vaccination” within communities, in order to be make vaccination programmes understandable, acceptable and therefore sustainable [5,6]. There is still much to do in order to promote vaccine empowerment and increase parents’ knowledge, abilities and skills, enabling them to make aware choices [7,8,9]. A small part of the population is firmly opposed to all vaccines, while about 20–30% has doubts and concerns that may lead to delay in acceptance/refusal of some vaccines or to follow the vaccine schedule with reluctance [10,11].

Parents’ attitude towards vaccination is affected by several elements linked to confidence, convenience, and complacency [10]. Knowledge on vaccines is one of the factors that affect parents’ decision-making process, and consequently communication is one of the strategies that can contribute to address vaccine hesitancy. Parents’ knowledge about vaccination results from complex, and not always conscious, interactions of information from different sources, not only the recommendation/advice of healthcare providers, but also the opinions of family members and friends, media, websites, online social networks, and ideas arising from direct or indirect experiences. The internet and the media are widely used by anti-vaccinists to amplify scientifically unfounded information about vaccines (e.g., the presence of heavy metals) and thus favouring scepticism and mistrust about immunisation [11,12,13,14,15,16,17]. Overall, parents receive several messages regarding vaccines, often even conflicting, they interpret them subjectively, and give them an overall meaning that, even if it is not supported by scientific evidence, will guide their choices [18,19]. In the age of globalisation, public health services cannot prevent different stakeholders from disseminating news without scientific evidence, but they have the duty to strengthen their ability to communicate effectively regarding vaccines. No opportunities should be missed in order to meet parents’ needs and spread the culture of vaccination [2,20,21].

Communicating effectively about vaccines does not simply mean providing technical information or counteracting false information according to ineffective persuasive models [7,8]. It means implementing interactive processes based on the exchange of information, knowledge, needs, attitudes, and emotions between the involved parties. Additionally, communicating effectively means identifying settings where parents can be more receptive to the issues of vaccines [22,23]. The literature suggests the importance of early settings, in which communication interventions are carried out in a temporal context that precedes the administration of vaccines, leaving parents time to consider, discuss and reflect on the received information [24,25,26,27]. Prenatal courses and birth points can be considered “early settings” in which the communication regarding vaccines is carried out through interactive methods during pregnancy or within the days of delivery, i.e., at least two–three months before the first session of vaccination. 

In countries such as Italy, almost all births take place in hospitals and prenatal courses are offered free of charge by local health services. However, the communication regarding vaccines is not an integral part of the institutional mission of these services, and the implementation of vaccination meetings during the antenatal course or at the point of birth depends on the resourcefulness of the single health workers of these services, and is not widespread in the national territory [28]. The hypothesis of an association between vaccine attitude and exposure to interactive meetings in an early setting, if supported by several studies, could trigger further studies with experimental designs, leading to institutionalising meetings on vaccines in prenatal courses and at birth centres. 

The present paper aims to contribute to the assessment of this hypothesis by presenting the results of an explorative “natural history” study that analyses the attitudes of a sample of Italian mothers of new-borns towards vaccines and its potential association with interactive meetings on this topic carried out during prenatal courses and at birth points.

## 2. Methods

### 2.1. Context of the Study

This study was performed in Cagliari, Sardinia (Italy) between 2015 and 2016. During these years, the law no.119/2017 on compulsory vaccination had not come into force yet; the National Health System (NHS) provided free-of-charge childhood vaccination, which includes four compulsory vaccines (poliomyelitis, diphtheria, tetanus and hepatitis B), and eight recommended ones (pertussis, haemophilus, measles, rubella, mumps, chicken pox, meningococcal group c, pneumococcal disease).

### 2.2. Design of the Study

This exploratory study was carried out during a broader cross-sectional study mainly aimed at analysing informative needs and attitudes towards breastfeeding. In order to collect data, a questionnaire in the Italian language was administered through structured interviews by trained health professionals. Data were collected anonymously. 

### 2.3. Recruitment of Participants

Participants were recruited among mothers of new-borns waiting for congenital hip dysplasia ultrasound screening to be taken in one of the regional dedicated centres in Cagliari; the regional health system offers this screening to all new-borns. A hip ultrasound is performed in the first period of the child’s life, on average at 1–2 months. During the recruitment phase, the researchers explained the following aspects: participation in the survey was voluntary, no identifying data would be collected, the interview would focus on knowledge and attitudes towards breastfeeding and vaccination, the results of the survey will be published only in aggregate form. In accordance with the research topic and method, we considered verbal consent appropriate. Asking for verbal consent and administering the questionnaire by interview was the ideal way to encourage participation, because it allowed the interviewees to participate in the survey and at the same time to care for their new-born babies (e.g., cradle them, breastfeed them). Less than 5% of mothers refused to undertake the interview (*n* = 12). In order to minimise risks of recall bias, only mothers of children younger than 4 months of age were involved in the study. In total, 266 mothers were interviewed, and 95% of new-borns were less than three months old. 

### 2.4. Questionnaire

The first part of the questionnaire was aimed at collecting social and demographic data, as well as some variables concerning attendance of prenatal courses, whether they were primigravidae and how old their babies were in that moment. In order to assess exposure to information on vaccines during pregnancy and childbirth, and attitude towards vaccines, the following questions were asked:

*Did you receive or seek information on vaccines during pregnancy /childbirth? Consider both health and non-health sources.*

(yes or no)

*During the pre-natal course and/or at birth point did you have the opportunity to participate in small group meetings (about 8–10 people), conducted in a participatory way (not frontal lessons, but interactive meetings where you could express yourselves freely), and focused on the topic of vaccines (vaccination schedule, benefits and risks)?*

(yes or no)

*- If you had the opportunity to participate in these meetings, was it useful?*

(yes or no)

*- If you did not attend these meetings, would you have been glad to have this opportunity?*

(yes or no)

*- Are you going to submit your child to the free vaccinations offered by the National Health System, within the proposed timetable?*

(open ended question)

*- If you are not going to submit your child to all the free vaccinations offered by the National Health System, which vaccines are you more hesitant about and why?*

(open ended question)

In the open ended questions, the interviewers reported the exact words used by the interviewees.

**Figure 1:**
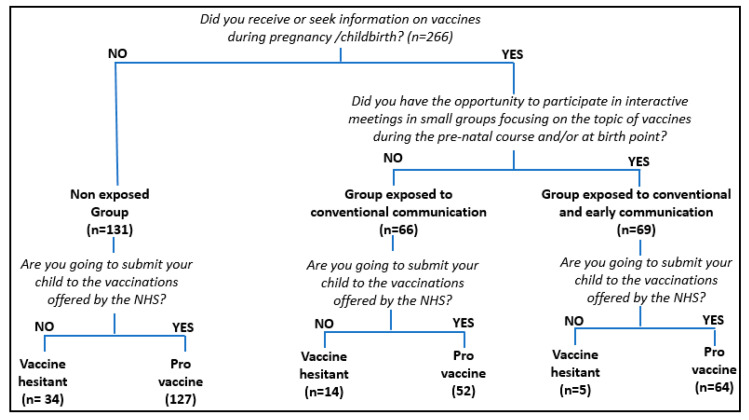
Interview process and data extraction process.

The interview process and data extraction process are shown in Figure 1.

As for the different exposures to communication regarding vaccination, mothers were classified into three groups:

(1) *“Non-exposed group*”, i.e., mothers who declare that they have not received or looked for any specific information on vaccines during pregnancy/childbirth and who have not attended meetings on this topic.
(2) *“Group exposed to conventional communication”,* i.e., mothers who declare that, during pregnancy/childbirth, they have received and/or looked for information about vaccines by means of healthcare and non-healthcare sources, but who have not attended meetings on this topic.
(3) “*Group exposed to conventional and early communication*”, i.e., mothers who declare that they have received or looked for information about vaccines by means of healthcare and non-healthcare sources, and who have attended meetings about vaccines during the prenatal course and/or at birth point.

As for the attitudes to vaccinations, mothers were asked whether their children would undertake all the free-of-charge vaccinations offered by the National Health System (NHS). Attitudes to vaccinations were classified according to the following definitions:

(1) *Pro-vaccine attitude*, i.e., mothers who steadily declare that they would follow the vaccination schedule offered by the NHS (both compulsory and recommended) without any doubt or concern.
(2) *Vaccine hesitancy attitude*, i.e., mothers who express strong perplexity and reluctancy and/or declare that they would not entirely follow the vaccination schedule proposed by the NHS.

### 2.5. Data Analysis

Collected data were analysed by means of descriptive statistics with 95% confidence intervals. A chi-squared test for linear trend was used to analyse the association between the attitude to vaccination and the different exposures to communication regarding vaccines. Additionally, this association was analysed through a multivariable logistic regression model in order to assess potential confounding effects of the main social and demographic variables. In the logistic model, vaccination attitude was included as the dependent variable (vaccine hesitancy attitude vs pro-vaccine attitude). Age of mothers (continuous variable), education (having a diploma or more vs lower education level), being primigravidae (yes vs no), working in the health sector (yes vs no), premature delivery (yes vs no) and exposure to information on vaccines (non-exposed mothers vs mothers exposed to conventional and early communicative settings; mothers exposed to conventional communicative setting vs mothers exposed to conventional and early communicative settings) were included as independent variables. Starting from the saturated model including all independent variables, non-significant associated variables (*p* value ≥ 0.05) were consecutively deleted from the model through a step by step procedure managed by researchers. The goodness of fit of the models obtained in each step was assessed through the likelihood statistics. The final regression model included only significant associated variables. The estimated sample size necessary to assess factors associated with vaccination attitude was 250.

In order to explore the reasons for the attitude towards vaccine hesitancy, the answers to the open-ended question on this topic were analysed, merging similar contents into the same category. The results were illustrated through a mental map, with a textual explanatory quotation for each identified category.

## 3. Results 

### 3.1. Sample Description

The study involved 266 mothers of new-born babies who gave birth to their child less than 4 months ago. Sample description is reported in Table 1.

At the moment of the study, 67% of the babies were 1 or 2 months old. The mean age for the mothers was 33 years, about 3/4 of them declared to live in the metropolitan area of Cagliari, 81% completed high school, 4% work in the health field, 62% were primigravidae and 39% attended a prenatal course. Lack of time, already having other children and lack of interest were the reasons for not attending the prenatal course.

Overall, 26% (*n* = 70) of the interviewed women participated in interactive meetings on vaccines during attendance of the prenatal classes or at the birth point.

### 3.2. Attitude to Vaccination

As far as attitude towards vaccination is concerned, 80% of mothers (CI 76–84%) said, without any uncertainty, that they were willing to follow the whole vaccine schedule provided by the NHS, and to respect the recommended deadlines; the remaining 20% (CI 16–24%) showed an orientation towards vaccine hesitancy. More specifically, 48 out of 53 mothers stated that they would like to submit their children only to some of the free vaccinations offered by the NHS, while the remaining five mothers said they would postpone joining the vaccination program.

Mothers showing vaccine hesitancy were asked, by means of an open-ended question, which vaccines they more hesitant about: 53% generically referred to all the vaccines without of any specific mention, 30% expressed hesitancy about the recommended vaccines without any specific mention, while only 17% mentioned specific vaccines. More specifically, 13% of hesitant mothers cited vaccination against measles–mumps–rubella, 2% meningococcal vaccination and 2% tetanus.

**Figure 2:**
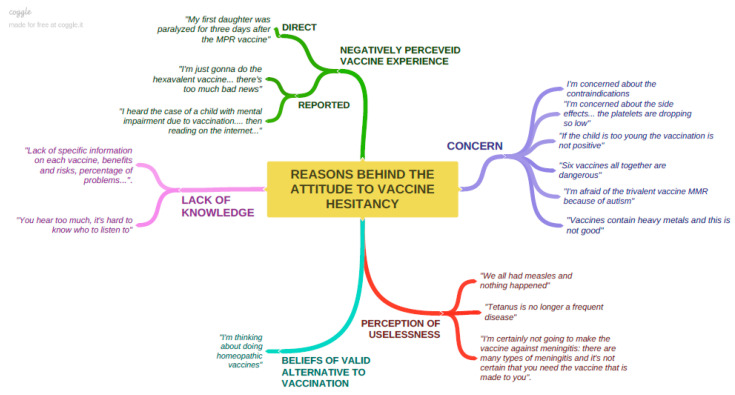
Reasons for the attitude to vaccine hesitancy.

As for the reasons for the attitude to vaccine hesitancy, almost all the “vaccine-hesitant” mothers declared to be worried about possible negative consequences linked to vaccines (Figure 2).

The stated reasons for concern were contraindications and side effects, the low age for vaccination, vaccine ingredients, the number of suggested vaccines per session (considered as the cause for an excess in immune stimulation), and risk for autism. Additionally, negatively perceived vaccine experiences contributed to determining concern, not only if they have been experienced in a direct way, but also when they are simply reported. Concern about the potential consequences of vaccines was sometimes coupled with the perceived futility of some vaccines; this perception emerged both in relation to the perceived non-hazardousness (measles) or low frequency (tetanus) of some diseases, and in relation to the inability of some vaccines to provide protection against all possible serotypes capable of transmitting a specific disease (meningitis). The perception of not having acquired the necessary knowledge to be able to decide was an additional factor of vaccine hesitancy; this perception was linked to the co-existence of conflicting information and to the feeling of not having received all the necessary information on risks and benefits of vaccination. The belief of valid alternatives to vaccination was reported by one interviewee, with specific reference to “homeopathic vaccines”.

### 3.3. Exposure to Information on Vaccines 

Results on different exposures to communication regarding vaccines are reported in Table 2. 

Nearly half of the respondents (49%) were included in the “*Non exposed group*”, as they did not remember having received or looked for any specific information about vaccination during pregnancy/childbirth; 25% of mothers were included in the “*Group exposed to conventional communication*”, as they stated they received or looked for information from several health and non-healthcare sources, but they did not take part in specific meetings during the antenatal classes or at birth centres; 26% of mothers were included in the “*Group exposed to conventional and early communication*”, as they declared having received or looked for information by means of healthcare and non-healthcare sources, as well as having taken part in interactive meetings in small groups during antenatal classes or at birth centres.

Among mothers who attended meetings on vaccines, 94% stated that it was a useful experience; among those who did not attend specific meetings, 89% would have appreciated taking part in them. It is noteworthy that among the “vaccine-hesitant” mothers who did not attend specific meetings on vaccines during the antenatal classes or at birth centres, 87% would have appreciated taking part in them.

### 3.4. Association between Attitude to Vaccination and Different Exposures to Communication Regarding Vaccines

The chi-squared test for linear trend indicated that attitude towards vaccine hesitancy tends to reduce as exposure to different communicative settings increases (Table 3).

Vaccine hesitancy reached 26% among mothers who did not remember having received or looked for information; it slightly decreased in mothers who declared having received information by means of different sources (21%), and it showed a considerable decrement in mothers who declared having taken part in specific meetings during antenatal classes or at birth centres (7%).

The multivariable logistic regression model showed that socio-demographic variables were not significantly associated with the attitude towards vaccination and they did not show confounding effects; therefore, they were deleted from the model. The odds ratio (OR) values, 95% confidence intervals (CI) and *p* value of the deleted variables are as follows: premature delivery (OR = 0.99; CI 95% 0.44–2.21; *p* = 0.98), being primigravidae (OR = 1.06; CI 95% 0.54–2.01; *p* = 0.89), working in the health sector (OR = 0.68; CI 95% 0.07–6.3; *p* = 0.73); living in Cagliari metropolitan area (OR = 1.40; CI 95% 0.62–3.18; *p* = 0.42), having a diploma or more education (OR = 1.54; CI 95% 0.63–3.8; *p* = 0.34); age of mothers (OR = 1.04; CI 95% 0.99–1.10; *p* = 0.15). Significantly associated variables are reported in Table 4.

Exposure to different communication settings was the only variable significantly associated with attitude towards vaccination. Considering the group of the mothers who were exposed in the early setting as the reference group (OR = 1), the risk for vaccine hesitancy was tripled for mothers exposed to a conventional communicative setting (OR 3.5) and it was quadrupled for mothers who were not exposed to any settings (OR = 4.5).

## 4. Discussion

This explorative study provides an overview of the vaccination attitudes of mothers of new-borns in Sardinia (Italy), and of its association with previous exposures to different communication settings, and thus contributing to detecting useful clues for triggering experimental studies on strategies for communicating effectively on this relevant topic.

In agreement with other studies, the results show that the attitude towards vaccine hesitancy affects about one-fifth of new-born mothers, who express doubts and worry more than totally rejecting all vaccines; this is mainly attributable to the combination of concern and perception of lack of knowledge, in line with other studies [1,11,28,29]. These findings confirm the specific needs that need to be addressed to communicate effectively about early childhood vaccinations. Not only balanced information about both the benefits and risks of vaccinations, but also support for the management of parents’ concern [11,13,25,30]. This concern, that in vaccine-hesitant mothers seems to be an important criterion in the subjective process of risk/benefit assessment of vaccinations, cannot be ignored or diminished. To promote vaccination empowerment, concern must be accepted and understood in depth, only in this way is it possible to give an adequate response to the emotional component and to the underlying cognitive needs, and to support mothers in the conscious decision-making process regarding the vaccination of their children [11,21,25,27].

Additionally, our results support the hypothesis that it could be useful to strengthen institutional communication regarding vaccines in those settings where it is possible to use the interpersonal channel with health professionals. Interpersonal communication could facilitate a process of participatory, empathic communication, based on the exchange of information, knowledge, needs, attitudes, and feelings between the subjects involved [11,13,23,25,28]. It is noteworthy that the usefulness of interpersonal communication with health professionals was confirmed by almost all the interviewed vaccine-hesitant mothers who did not take part in any meetings taking place in early settings; indeed, they declared that they would be glad to have this opportunity.

This result suggests that the need for interpersonal communication regarding vaccination is relevant in the case of concerns and doubts about vaccines, and confirms the need to make institutional communication regarding vaccines more widespread, expanding the contexts in which it is possible to have face to face communication with health professionals [13,14,31]. The present study supports the hypothesis that participation in interactive meetings in small groups focused on vaccination during the prenatal course or at the point of birth may act as an enabling factor. This can lead to a decrease in the tendency to experience vaccine hesitation [11,25,32,33]. It is well known that the effectiveness of a communication process also depends on the setting in which the communication is carried out, it is important to identify places, times and mental states in which the recipients are most receptive [25]. Earlier communication could facilitate message reception, thanks to the iterative process, through which evidence-based messages are transmitted, listened to, appraised, compared with messages from other sources, re-discussed, and finally, evaluated according to the same meaning they have for those who issued it [2,23,24,25,26]. It is reasonable to suppose that the communication carried out as part of the prenatal course or at point of birth is associated with a decrease in the tendency to experience vaccine hesitation, not only due to the use of the interpersonal channel, but also due to the timing of communication—about three months before the first vaccine session.

Our results seem to suggest that interactive meetings in early settings could play a valuable role in facing doubts and concerns that may lead mothers to delay/reject specific vaccines [1,11]. Further experimental studies are needed in order to deepen these results and investigate which features of the meetings on vaccines during the prenatal course or at birth point could help in reducing this trend.

This study is subject to some potential limitations. A selection bias due to the method used for sample recruiting cannot be excluded, although the terms of payment for hip ultrasound scan (co-payment or free of charge if tax exemption occurs) minimizes this risk; other studies show a similar percentage of vaccine-hesitant mothers, so there is a limited possibility that this bias could have occurred. We cannot exclude the possibility that mothers who attended meetings during prenatal classes or at birth point could be naturally more vaccination-oriented; however, 87% of vaccine-hesitant mothers who did not attend specific meetings in early settings declared that they would have been glad to have this opportunity. A further limitation is represented by the fact that the study does not involve the fathers of new-borns, although the literature suggests a preponderant role of the mother figure in vaccination choices for children.

On the whole, this study confirms that both concern and lack of knowledge contribute to trigger vaccine hesitancy in about 20% of new-born mothers. It also shows that the participation in interactive meetings on vaccination during the prenatal course or at birth point could favour a decrease in vaccine hesitancy. These results support the hypothesis that implementing communication regarding vaccines in early settings through interpersonal communication with healthcare staff and peers could be a resource to promote conscious adherence to vaccination. Further experimental studies are needed.

Our outcomes are in line with multiple studies that emphasize the strategic role that antenatal settings can play in vaccine communication. These studies bring to light the importance of starting to address the issue of childhood vaccinations during pregnancy in order to support and accompany future mothers towards thoughtful and proper decision-making [22,23,24,25,26,27,34,35,36,37,38]. In a recent survey conducted in Italy, vaccine hesitancy did not appear to be influenced by communication in antenatal settings [28]. However, we cannot overlook the fact that our assessment of exposure to communication (i.e., participation in interactive meetings) differed from the ones used in that study (i.e., receiving information). Consequently, it is reasonable to hypothesise that the difference between the results of the two studies on the role of communication in the antenatal setting may be affected by the different definition of the variable under study. Moreover, methods for data collection in our study were based on interviews with mothers of children younger than 4 months of age, while in the other study was based on a self-administered questionnaire with parents of children between 16 and 36 months of age; it is not possible to exclude that recall bias had a different weight in the two studies, and it could also influence the evaluation of the association differently. According to several studies, the antenatal period is a crucial phase where it is possible to help parents to deepen and consolidate their knowledge of vaccines; there is some evidence of the importance of highlighting not only the merits of vaccinations for the protection of children’s health, but also of fully understanding and meeting parents’ concerns about the safety of vaccines. However, the specific information and approaches that are effective in communicating knowledge on vaccines with future mothers are not yet clearly defined. Further studies should focus on building a framework that defines the essential features for implementing communication intervention in the antenatal period.

## 5. Conclusions

This study suggests some implications that could be not only relevant for Italy, but also for other countries with a similar health system and social-economic contexts. According to the World Health Organization (WHO) “*People who delay or refuse vaccines for their children are presenting a growing challenge for countries seeking to close the immunization gap*” [22]. The National Health System could begin to address this challenge by using the available resources in an effective and efficient way. Concern and perceived lack of information play a role in vaccine hesitancy, while communication regarding vaccines implemented in early settings through interpersonal channels could be an enabling factor for vaccine adherence. Considering that prenatal courses and birth points are already active across the national territory, including communication regarding vaccinations among the regular activities of these services could be a proper strategy.

## Figures and Tables

**Table 1:** Description of the sample.

Parameters	*n*	Estimates (95% CI)
Mothers’ mean age	266	33 (32–33)
Mothers living in Cagliari metropolitan area	203	76% (71–81%)
Mothers having a diploma or being graduated	216	81% (77–86%)
Mothers working in the healthcare sector	6	4% (2–7%)
Primigravidae	164	62% (57–67%)
Mothers having attended prenatal classes	106	39% (34–45%)

**Table 2:** Different exposures to communication regarding vaccines: prevalence of mothers in each subgroup.

Exposure to Communication Regarding Vaccines	Prevalence (95% CI)
Non-exposed group *Mothers who declare that they have not received or looked for any specific information on vaccines during pregnancy/childbirth and who have not attended meetings on this topic (n = 131).*	49% (41–58%)
Group exposed to conventional communication *Mothers who declare that they have received and/or looked for information about vaccines by means of healthcare and non-healthcare sources, and who have not attended meetings on this topic (n = 66).*	25% (14–35%)
Group exposed to conventional and early communication *Mothers who declare that they have received or looked for information about vaccines by means of healthcare and non-healthcare sources, and who to have attended meetings about vaccines during prenatal course and/or at birth point (n = 69).*	26% (16–36%)

**Table 3:** Prevalence of vaccine hesitancy in mothers with different exposures to communication regarding vaccine.

Exposure to Communication Regarding Vaccines	Prevalence of Vaccine Hesitancy % (95% CI)	*p* Value
Non-exposed group *Mothers who declare that they have not received or looked for any specific information on vaccines during pregnancy/childbirth and who have not attended meetings on this topic (n = 131).*	26% (18–33%) *n* = 34	0.002
Group exposed to conventional communication *Mothers who declare that they have received and/or looked for information about vaccines by means of healthcare and non-healthcare sources, but who have not attended meetings on this topic (n = 66).*	21% (11–31%) *n* = 14	
**Group exposed to conventional and early communication** *Mothers who declare that they have received and/or looked for information about vaccines by means of healthcare and non-healthcare sources, and who have attended meetings on this topic (n = 69).*	7% (1–13%) *n* = 5	

**Table 4:** Results of multivariable logistic regression model: variables significantly associated with vaccine hesitancy attitude.

Independent Variables	OR (CI 95%)	*p* Value
Non-exposed mothers (*n* = 131)	4.5 (1.7–12.1)	<0.001
Mothers exposed to conventional communication (66)	3.4 (1.2–10.2)	0.03
Mothers exposed to conventional & early communication (*n* = 69)	1 (reference)	0.012

## Data Availability

The dataset used during the current study is available from the corresponding author upon reasonable request.

## Abbreviations

WHO	World Health Organization
NHS	National Health System
OR	odds ratio
CI	confidence interval
